# Prognostic value of presepsin in sepsis and septic shock: a meta-analysis

**DOI:** 10.3389/fimmu.2025.1680877

**Published:** 2025-10-15

**Authors:** Xiaokang Xing, Qianwen Wang, Yueyue Zhang, Ge Zhang

**Affiliations:** Department of Critical Care Medicine, Sir Run Run Shaw Hospital, Zhejiang University School of Medicine, Hangzhou, Zhejiang, China

**Keywords:** presepsin, sepsis, septic shock, prognosis, meta-analysis

## Abstract

**Background:**

Presepsin, an innate immune activation biomarker, shows potential for predicting the prognosis of sepsis, but its predictive accuracy remains unclear. This meta-analysis aims to evaluate its efficiency for predicting the risk of mortality in sepsis and septic shock.

**Methods:**

Per PRISMA guidelines, four databases were searched until January 2025. Sixteen observational studies with 2,066 patients were included. Pooled sensitivity, specificity, DOR, and AUC were calculated using bivariate random-effects models. The sources of heterogeneity were explored via subgroup analyses and meta-regression. Study quality was assessed with QUADAS-2.

**Results:**

Presepsin showed moderate accuracy for predicting the risk of mortality (pooled AUC 0.80, 95% CI: 0.76-0.83). The pooled sensitivity and specificity were 76% (95% CI: 69-82%) and 70% (95% CI: 59-78%), respectively. Sensitivity was notably higher in patients with septic shock (90%) compared to those with sepsis (75%), whereas specificity was comparatively lower (50% vs. 77%). Substantial heterogeneity stemmed from threshold variability and geographic differences (particularly in Korean cohorts). Prospective studies had higher sensitivity (80%) than retrospective studies (60%). No publication bias was detected.

**Conclusions:**

Presepsin is a pragmatic biomarker for predicting the risk of mortality, with enhanced sensitivity in septic shock. Presepsin may be integrated into clinical decision-making for early intervention.

**Systematic Review Registration:**

PROSPERO, identifier CRD42025639268.

## 1 Introduction

Sepsis is a complex clinical syndrome characterized by potentially fatal multi-organ dysfunction resulting from a dysregulated host response to infection (1). Prompt and accurate identification of sepsis is of paramount importance, as it facilitates the early initiation of targeted therapeutic interventions that substantially influence patient survival and clinical outcomes (2). Central to this objective is the identification of pathognomonic biomarkers that are selectively elevated in the early stages of sepsis pathogenesis and possess robust predictive validity, thereby optimizing the therapeutic window in critically ill populations.

Currently, several biomarkers have been validated for the diagnosis of sepsis, with procalcitonin (PCT) and C-reactive protein (CRP) being among the most extensively utilized. Despite their widespread clinical adoption, these biomarkers exhibit notable limitations (3). CRP, an acute phase protein, can be elevated in a variety of non-infectious inflammatory conditions, including trauma, surgery, burns, and autoimmune diseases, significantly reducing its specificity in distinguishing infectious from non-infectious inflammatory conditions (4). Similarly, PCT can be abnormally elevated in non-infectious systemic inflammatory response syndrome (SIRS), severe trauma, burns, major surgery, and certain specific treatments (5). CRP has a half-life of approximately 19 hours, and it begins to rise 6–12 hours after the onset of infection, reaching its peak 24–48 hours later. Given this delayed effect, its effectiveness in early diagnosis is limited (6). In contrast, although PCT begins to rise 3–6 hours after infection, its levels are significantly affected by renal function. Multiple studies have shown that CRP and PCT levels are not significantly correlated with 28-day mortality in patients with sepsis (7, 8). Large randomized controlled trials have shown that PCT-guided treatment plans fail to improve patient outcomes compared with traditional clinical assessments and may instead prolong ICU stay and increase the need for mechanical ventilation (5). Therefore, the identification of more reliable biomarkers and advanced diagnostic methods remains an urgent unmet need in sepsis management.

Presepsin, also termed soluble CD14 subtype, is a 13-kilodalton N-terminal fragment generated through proteolytic cleavage of the CD14 glycoprotein, released into the circulation following activation of host inflammatory signaling pathways in response to pathogenic challenge (9). Since its initial identification in 2005, presepsin has emerged as a pathophysiologically relevant biomarker, exhibiting significant elevation in septic patients (10). Compared with traditional markers such as procalcitonin (PCT) and C-reactive protein (CRP), presepsin increases significantly earlier after the onset of infection (11). This early response makes it particularly suitable for rapidly identifying high-risk patients in the emergency department, providing clinicians with a valuable window of time for treatment. Beyond diagnosis, presepsin also demonstrates excellent prognostic value. Studies have shown that presepsin levels correlate with disease severity and can be used to stratify the risk of mortality (11). Compared with other infection markers, presepsin is superior in predicting adverse outcomes (12). Presepsin, a soluble fragment of CD14, is primarily elevated under infectious conditions, with high specificity. In contrast, CRP and PCT can also be elevated in non-infectious inflammatory states. Given the specificity, presepsin may be more effective in distinguishing between infectious and non-infectious inflammatory conditions (13).

(9–13)Despite growing evidence suggesting its prognostic utility, substantial uncertainty remains regarding the consistency of presepsin’s predictive performance. Previous studies have yielded heterogeneous findings; for instance, Hassan et al. reported strong predictive accuracy for in-hospital sepsis-related mortality (area under the curve (AUC) = 0.824), while Koh et al. observed considerably lower discriminatory capacity (AUC = 0.656) in their cohort (14, 15). These discrepancies underscore the need for a comprehensive meta-analytical approach to quantitatively synthesize available data, derive pooled estimates with statistical rigor, and explore potential sources of heterogeneity across studies.

This meta-analysis aims to evaluate the prognostic utility of presepsin in patients with sepsis and septic shock, determine optimal cutoff thresholds for mortality prediction, and evaluate its potential role in the early identification of high-risk patients. By integrating current evidence, our study seeks to present clinically meaningful insights into the incorporation of presepsin within sepsis risk stratification frameworks to guide timely interventions.

## 2 Methods

Before commencement, our study was registered with the PROSPERO database (Registration No.: CRD42025639268) and followed the Preferred Reporting Items for Systematic Reviews and Meta-Analyses of Diagnostic Test Accuracy Studies (PRISMA-DTA) guidelines (16).

### 2.1 Literature search

PubMed, EMBASE, the Cochrane Library, and Web of Science databases were systematically retrieved until January 10, 2025, with no restrictions on language. The search strategy incorporated the following Boolean operators: (‘presepsin’ OR ‘sCD14-ST’ OR ‘soluble CD14 subtype’) combined with (‘sepsis’ OR ‘septic shock’). The search strategy involved both controlled vocabulary (e.g., MeSH terms) and free-text keywords, interconnected through Boolean operators (OR/AND) to balance comprehensive coverage and precision. The complete search strategy is provided in Appendix 1.

### 2.2 Study selection

Studies were selected as per the PICOS framework: populations included adult patients (≥18) with sepsis or septic shock, all defined in accordance with the Third International Consensus Definitions for Sepsis and Septic Shock; exposure was presepsin (sCD14-ST) level measurement in serum/plasma with explicit cutoff thresholds; outcomes were mortality (e.g., 28-day, in-hospital) and diagnostic metrics (sensitivity, specificity, AUC, or 2×2 contingency tables); study designs were observational (prospective/retrospective cohort, case-control) peer-reviewed full-text articles. Exclusion criteria encompassed non-primary research, animal studies, non-sepsis populations, insufficient data, duplicates, and non-English articles without translations. Two independent reviewers (Xiaokang Xing and Qianwen Wang) systematically screened all retrieved records by title and abstract as per the predefined eligibility criteria. The full texts of potentially eligible studies were retrieved for final assessment by the same reviewers independently. Discrepancies arising at any stage were resolved through discussion or, if necessary, via consultation with a third reviewer (Ge Zhang).

### 2.3 Data extraction

Two investigators (Xiaokang Xing and Yueyue Zhang) independently extracted data using a standardized data collection form. Extracted information encompassed study characteristics (first author, publication year, country, design), demographic data (sample size, mean or median age, sex distribution), and relevant prognostic or diagnostic indicators (presepsin cutoff values, sensitivity, specificity, AUC, and mortality outcomes). For studies reporting multiple time points of presepsin measurement, we prioritized the baseline time point within 24 hours of sepsis diagnosis/admission (or the first detection time point labeled in the study). If a study had multiple baseline-related time points, we extracted the one closest to sepsis diagnosis (as per Sepsis-3 criteria). For missing or ambiguous information, efforts were made to reach out to the authors of the original study for clarification. Disagreements in data extraction were addressed via discussion or consultation with a third reviewer (Ge Zhang).

### 2.4 Quality assessment

The methodological quality of the included studies was rated via the Quality Assessment of Diagnostic Accuracy Studies-2 (QUADAS-2) tool (17), as implemented in Review Manager (RevMan 5.4; Cochrane Collaboration, Oxford, UK). Two reviewers (Xiaokang Xing and Yueyue Zhang) independently evaluated four key domains: patient selection, index test, reference standard, and flow and timing. The risk of bias in every study was classified as “low,” “high,” or “unclear” according to the following criteria: low risk: all signaling questions within each domain were answered “yes”; high risk: one or more questions were answered “no”; and unclear risk: the information provided was insufficient for a definitive judgment. Concerns regarding applicability were similarly evaluated and categorized. Any disagreements were settled through consensus or discussion with a third reviewer (Ge Zhang). Results were visualized via RevMan’s risk-of-bias graphs.

### 2.5 Statistical analysis

Statistical analyses were enabled by Stata 15.0 (StataCorp, College Station, TX, USA) and MetaDiSc 1.4 (Unit of Clinical Biostatistics, Ramón y Cajal Hospital, Madrid, Spain). The pooled sensitivity, specificity, and diagnostic odds ratios (DOR) with 95% confidence intervals (CIs) were derived via a bivariate random-effects model for between-study heterogeneity detection. The overall diagnosis accuracy of presepsin was visualized via summary receiver operating characteristic (SROC) curves. Heterogeneity was quantified via the I²statistic, with I²>50% denoting substantial heterogeneity. Possible sources of heterogeneity were examined through subgroup analyses and meta-regression. Subgroup analyses were conducted by disease type (sepsis vs. septic shock), study design (prospective vs. retrospective), geographic region, age (<65 vs. ≥65 years), and cut-off value (>1000 vs. ≤1000 pg/mL). The robustness of pooled estimates was rated via sensitivity analyses. The clinical utility of presepsin was assessed by illustrating pre-test and post-test probability relationships through a Fagan nomogram. Publication bias was detected via the Deeks’funnel plot asymmetry test, with P<0.05 suggesting significant bias. Each statistical test was two-tailed, and P<0.05 denoted statistical significance.

## 3 Results

### 3.1 Study selection and characteristics

**Figure 1:**
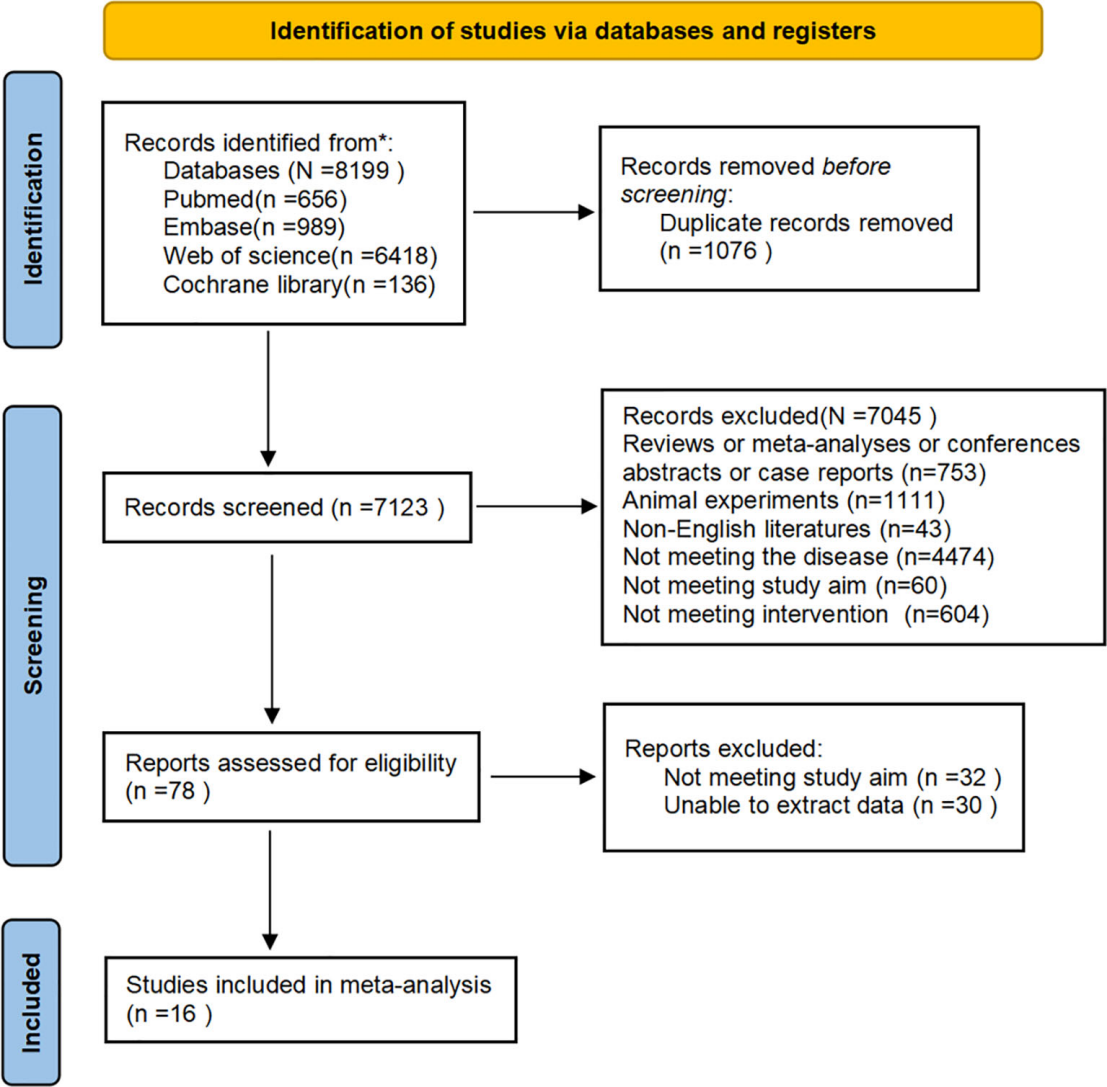
PRISMA flow chart.

Initial database searches yielded 8,199 records. After removing 1,076 duplicates, the titles and abstracts of 7,123 records were checked. Subsequently, the full texts of 78 articles were reviewed. Ultimately, 16 eligible studies were included in our meta-analysis. The process is presented in the PRISMA flow chart (**Figure 1**). Inter-reviewer agreement for study screening was assessed using Cohen’s Kappa coefficient (Kappa = 0.87), indicating excellent consistency.

This meta-analysis synthesized data from 16 clinical investigations published between 2016 and 2024, encompassing a cumulative total of 2,066 patients diagnosed with sepsis-related conditions. Among these, 1,549 patients were categorized as sepsis cases, and 517 had progressed to septic shock. The included studies were geographically distributed across six countries: Egypt (n=3), South Korea (n=6), China (n=3), India (n=2), Romania (n=1), and the Czech Republic (n=1). Concerning study design, 12 investigations adopted a prospective cohort methodology, while the remaining 4 were retrospective observational studies. The patient cohorts spanned a wide age range, with reported mean or median ages between 35 and 78. A detailed summary of baseline study characteristics is presented in **Table 1**.

**Table 1:** Baseline characteristics of the included studies.

Author	Year	Country	Design	Disease	Sample size (N)	Age	Cut-off value (pg/mL)	Sensitivity (%)	Specificity (%)	AUC
Abdelshafey	2021	Egypt	Prospective study	Sepsis	26	68.04 ± 18.60	640.00	100.00	66.67	0.920
Ali	2016	Egypt	Prospective study	Sepsis	33	55.20 ± 14.60	957.50	94.70	85.70	0.891
Baik	2022	Korea	Retrospective study	Sepsis	40	76.94 ± 17.95	1898.50	75.00	87.50	0.764
Brodska	2018	Czech Republic	Prospective study	Sepsis	30	65.29 ± 4.90	1843.00	75.00	75.00	0.734
Drăgoescu	2021	Romania	Prospective study	Sepsis	114	74.60 ± 8.49	2365.00	74.00	88.00	0.861
Hassan	2019	Egypt	Prospective study	Sepsis	68	35.70 ± 15.10	607.00	86.40	89.60	0.824
Juneja	2023	India	Retrospective study	Sepsis	70	62.80 ± 15.00	729.00	61.50	27.30	0.734
Kim	2017	Korea	Prospective study	Sepsis/Septic Shock	112/45	69.75 ± 3.77	2455.00	76.50	53.70	0.684
Koh	2021	Korea	Retrospective study	Sepsis	153	69.10 ± 14.00	1176.00	66.70	61.10	0.656
Lee	2022	Korea	Prospective study	Sepsis/Septic Shock	141/137	76.25 ± 3.58	821.00	68.90	50.50	0.605
Narendra	2022	India	Prospective study	Sepsis	92	50.07 ± 13.31	1466.00	77.40	81.00	0.856
Park	2021	Korea	Prospective study	Sepsis/Septic Shock	318	62.36 ± 15.00	755.00	77.50	62.00	0.747
Ren	2024	China	Prospective study	Septic Shock	285	72.05 ± 2.11	2553.50	92.20	39.60	0.661
Wen	2019	China	Prospective study	Sepsis	138	62.00 ± 4.21	2623.00	62.71	72.60	0.703
Wu	2023	China	Retrospective study	Sepsis/Septic Shock	164	62.84 ± 4.50	2232.55	53.80	75.30	0.634
Yang	2024	Korea	Prospective study	Septic Shock	50	78.24 ± 3.57	709.00	62.20	80.00	0.744

### 3.2 Quality assessment

**Figure 2:**
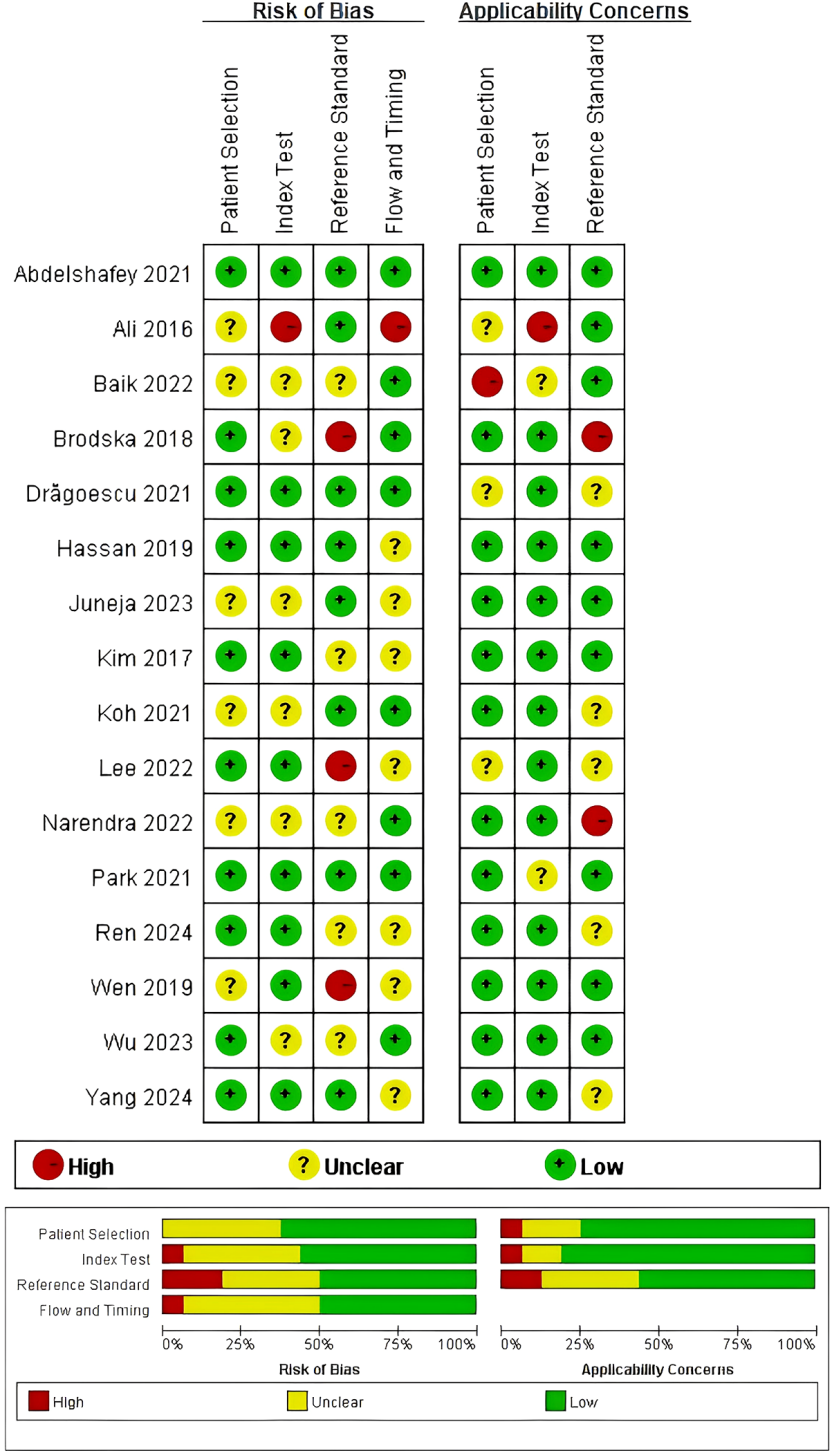
Summary of quality assessment.

The risk of bias and applicability concerns for the 16 eligible studies are displayed in **Figure 2**. Patient selection was deemed low risk in 10 studies and unclear in six studies. For the index test, nine studies demonstrated low risk, six were assessed as unclear, and one was considered high risk. Concerning the reference standard, eight studies exhibited low risk, five were unclear, and three were classified as high risk. In the domain of flow and timing, eight studies were rated as low risk, seven as unclear risk, and one as high risk. Concerning applicability concerns, patient selection posed low concern in 12 studies, unclear concern in three studies, and high risk in one study. For the index test, the concern is low, unclear, and high in 13 studies, two studies, and one study, respectively. Reference standard applicability was classified as low in nine studies, unclear in five studies, and high in two studies. The specific basis for the QUADAS-2 rating of each study in each domain is provided in Appendix 2.

### 3.3 Diagnostic performance

**Figure 3:**
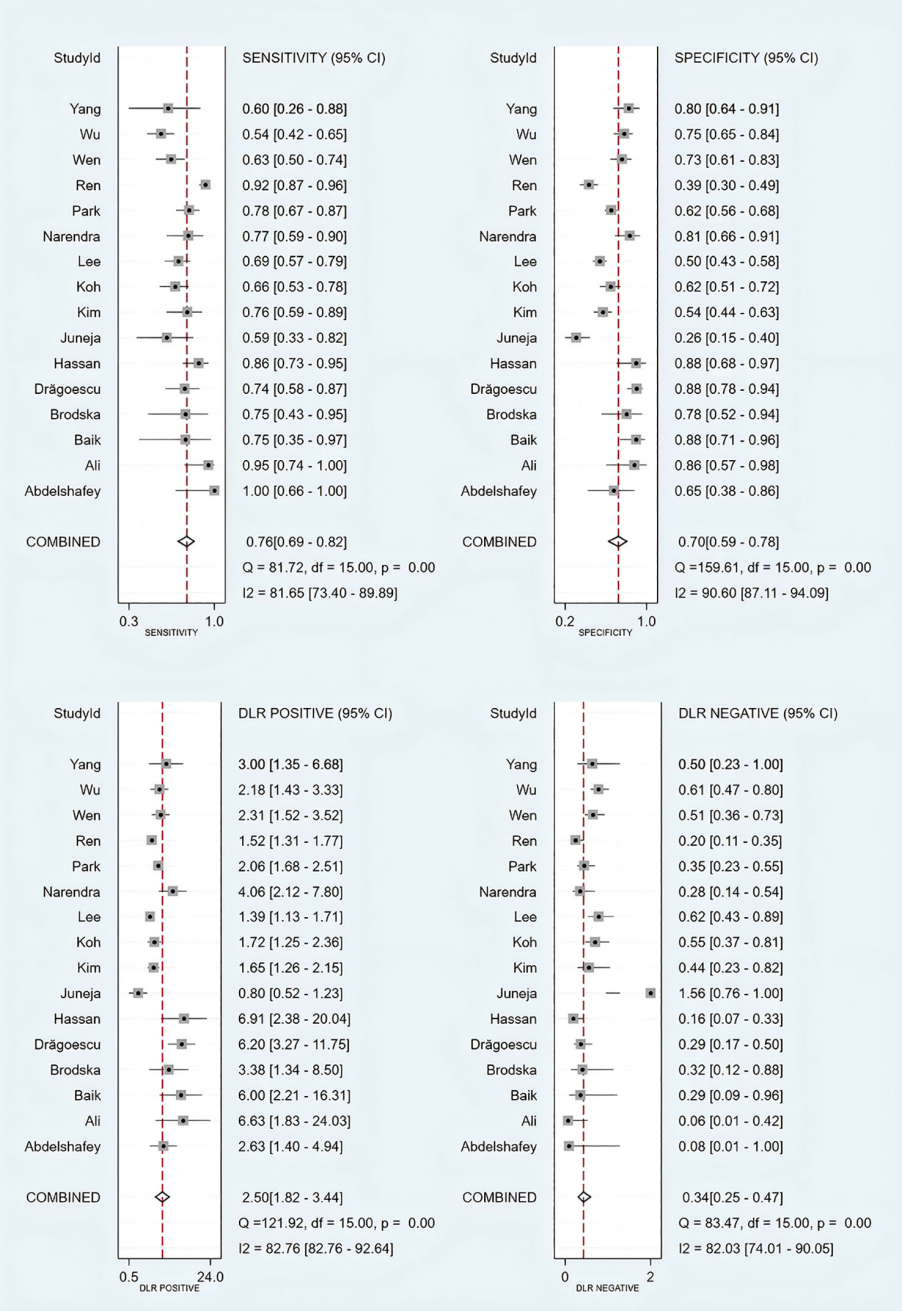
Forest plots of sensitivity, specificity, and positive and negative likelihood ratios of included studies.

**Figure 4:**
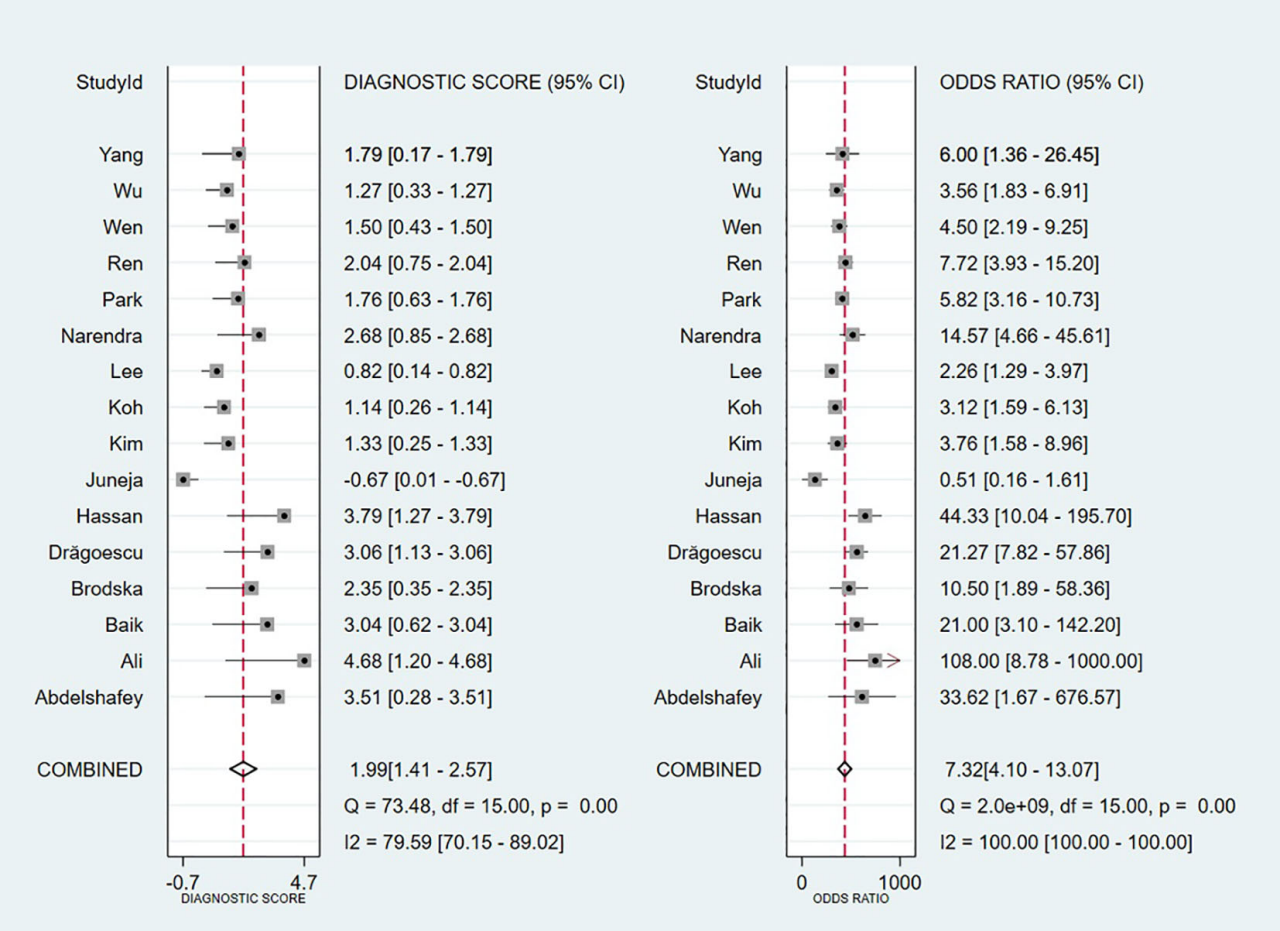
Diagnostic odds ratios for included studies.

**Figure 5:**
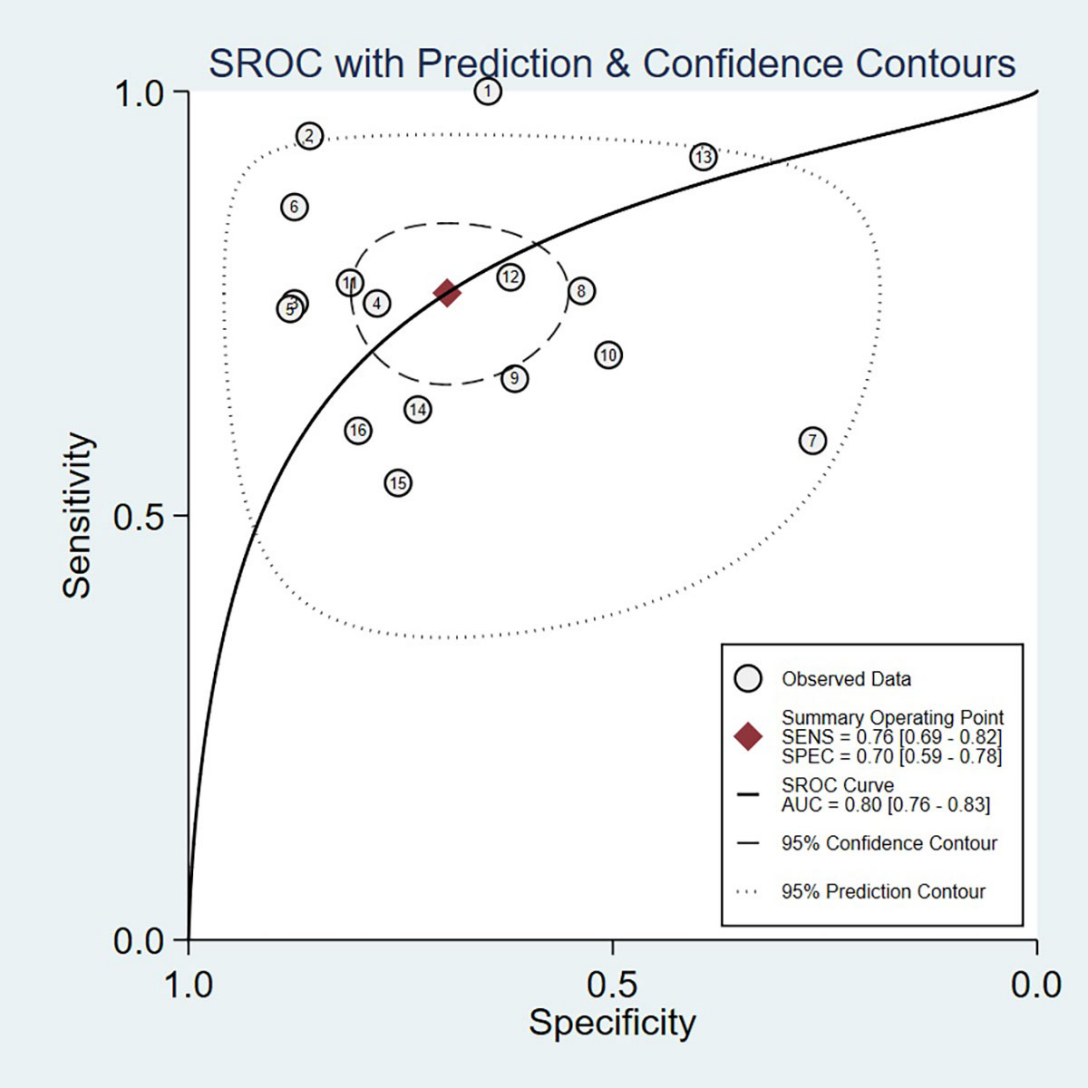
Summary receiver operating characteristic curves.

Threshold effect analysis via Spearman’s rank correlation coefficient demonstrated no significant threshold effect (coefficient = -0.149, P = 0.581). As depicted in **Figure 3**, the pooled sensitivity was 0.76 (95% CI: 0.69-0.82; I² = 81.7%) and specificity 0.70 (95% CI: 0.59-0.78; I² = 90.6%), with positive and negative likelihood ratios of 2.50 (95% CI: 1.82-3.44; I² = 82.8%) and 0.34 (95% CI: 0.25-0.47; I² = 82.0%), respectively. **Figure 4** presents the DOR (7.32; 95% CI: 4.10-13.07; I² = 100%). The SROC curve in **Figure 5** yielded an AUC of 0.80 (95% CI: 0.76-0.83), suggesting moderate diagnostic accuracy.

### 3.4 Subgroup analysis

Subgroup analyses were carried out by disease type, study design, geographical region, patient age, and diagnosis threshold (**Table 2**). In terms of disease subtype, pooled sensitivity was higher in patients with septic shock (0.90; 95% CI: 0.85-0.94) in comparison to those with sepsis (0.75; 95% CI: 0.66-0.82), while specificity exhibited an inverse pattern (0.50 vs. 0.77). Prospective studies demonstrated superior sensitivity (0.80; 95% CI: 0.72-0.86) relative to retrospective studies (0.60; 95% CI: 0.52-0.68), though specificity was relatively similar (0.71 vs. 0.65). Geographical stratification revealed that studies from Egypt exhibited the highest sensitivity (0.90; 95% CI: 0.81-0.96) and specificity (0.80; 95% CI: 0.67-0.90), followed by those conducted in China (sensitivity: 0.76; specificity: 0.59) and South Korea (sensitivity: 0.72; specificity: 0.65). Stratification by age demonstrated slightly higher sensitivity among older patients (≥65: 0.77 vs. <65: 0.74), albeit with lower specificity (0.68 vs. 0.72). Studies employing a diagnostic cut-off value >1000 reported comparable sensitivity (0.73 vs. 0.79) but improved specificity (0.72 vs. 0.67) in comparison to those using lower thresholds.

**Table 2:** Subgroup analyses of the prognostic performance of presepsin.

Subgroup	No of studies	Sen (95%Cl)	Spec (95%Cl)
Disease			
Sepsis	10	0.75 (0.66-0.82)	0.77 (0.64-0.86)
Septic shock	2	0.90 (0.85-0.94)	0.50 (0.42-0.58)
Design			
Prospective study	12	0.80 (0.72-0.86)	0.71 (0.60-0.79)
Retrospective study	4	0.60 (0.52-0.68)	0.65 (0.39-0.84)
Country			
Egypt	3	0.90 (0.81-0.96)	0.80 (0.67-0.90)
Korea	6	0.72 (0.64-0.78)	0.65 (0.54-0.75)
China	3	0.76 (0.71-0.81)	0.59 (0.53-0.65)
others	4	0.72 (0.60-0.81)	0.72 (0.43-0.89)
Age			
>65	9	0.77 (0.67-0.85)	0.68 (0.55-0.79)
<65	7	0.74 (0.61-0.84)	0.72 (0.55-0.85)
Cut - off value			
>1000	9	0.73 (0.63-0.81)	0.72 (0.60-0.81)
<1000	7	0.79 (0.69-0.87)	0.67 (0.49-0.81)

### 3.5 Meta-regression of heterogeneity sources

**Figure 6:**
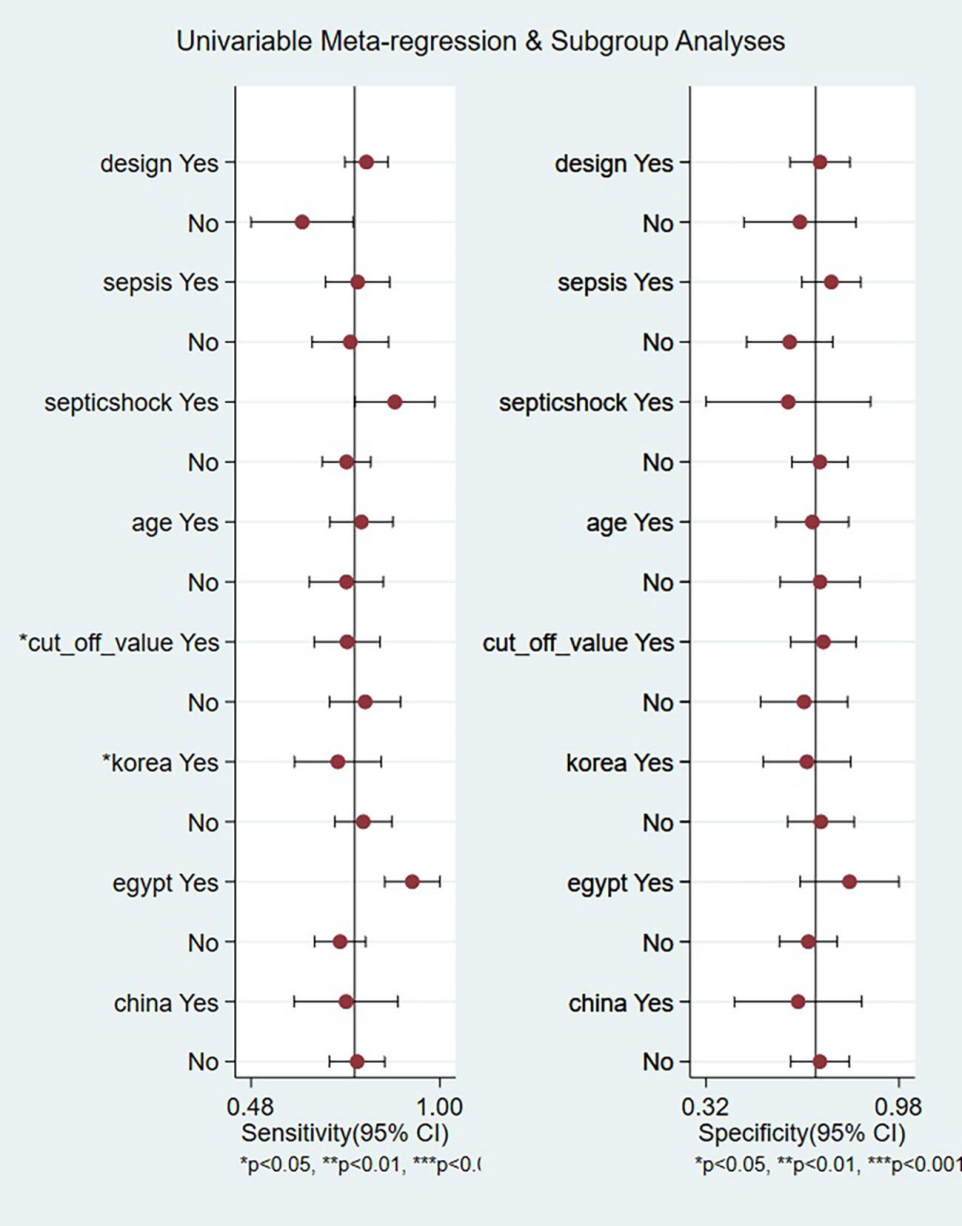
Univariable meta-regression and subgroup analyses identifying sources of heterogeneity in presepsin prognosis studies.

A meta-regression was conducted to investigate potential sources of heterogeneity, including study design, sepsis subtypes, age categories, diagnostic thresholds, and national cohorts (Korea, Egypt, China). As illustrated in **Figure 6**, two factors emerged as statistically significant contributors to the observed variation in sensitivity: differences in diagnostic threshold values (P < 0.05) and geographical variation pertaining specifically to Korean populations (P < 0.05). In contrast, no statistically significant associations with heterogeneity were identified for study design (prospective vs. retrospective), subgroups defined by sepsis versus septic shock, stratified age groups, or studies conducted in Egypt and China (all P > 0.05). These findings underscore the importance of threshold selection and regional characteristics within the Korean cohort as primary determinants of sensitivity variability, while other examined covariates did not substantially cause heterogeneity.

### 3.6 Clinical utility analysis

**Figure 7:**
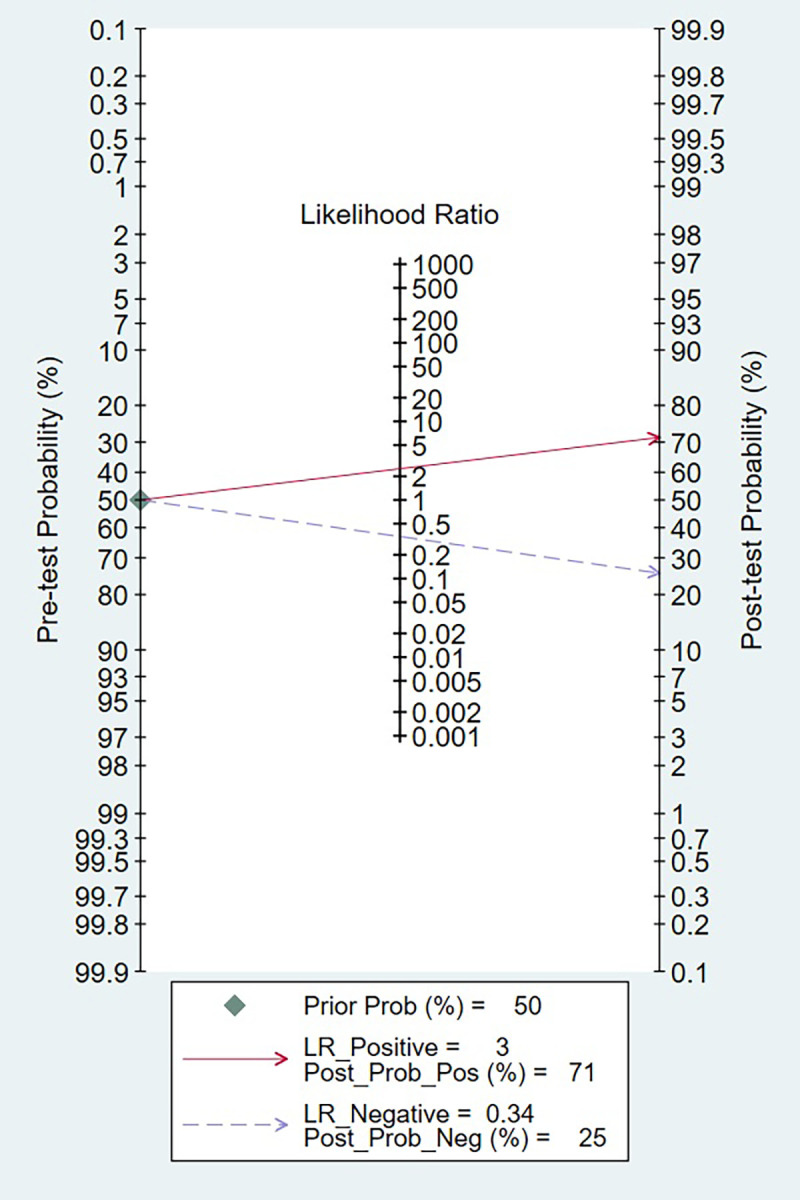
Fagan’s nomogram.

The clinical applicability of presepsin as a prognosis biomarker was evaluated using a Fagan nomogram, as depicted in **Figure 7**. Assuming a pre-test probability of 50%, a positive presepsin result increased the post-test probability of poor prognosis to 71%, whereas a negative result decreased it to 25%. These findings correspond to a positive likelihood ratio (LR+) of 3.0 and a negative likelihood ratio (LR−) of 0.34, thereby affirming the biomarker’s capacity to meaningfully alter post-test prognostic estimations. The absolute difference of 46% between positive and negative post-test probabilities highlights the substantial discriminative power of presepsin for clinical risk stratification.

### 3.7 Sensitivity analysis

**Figure 8:**
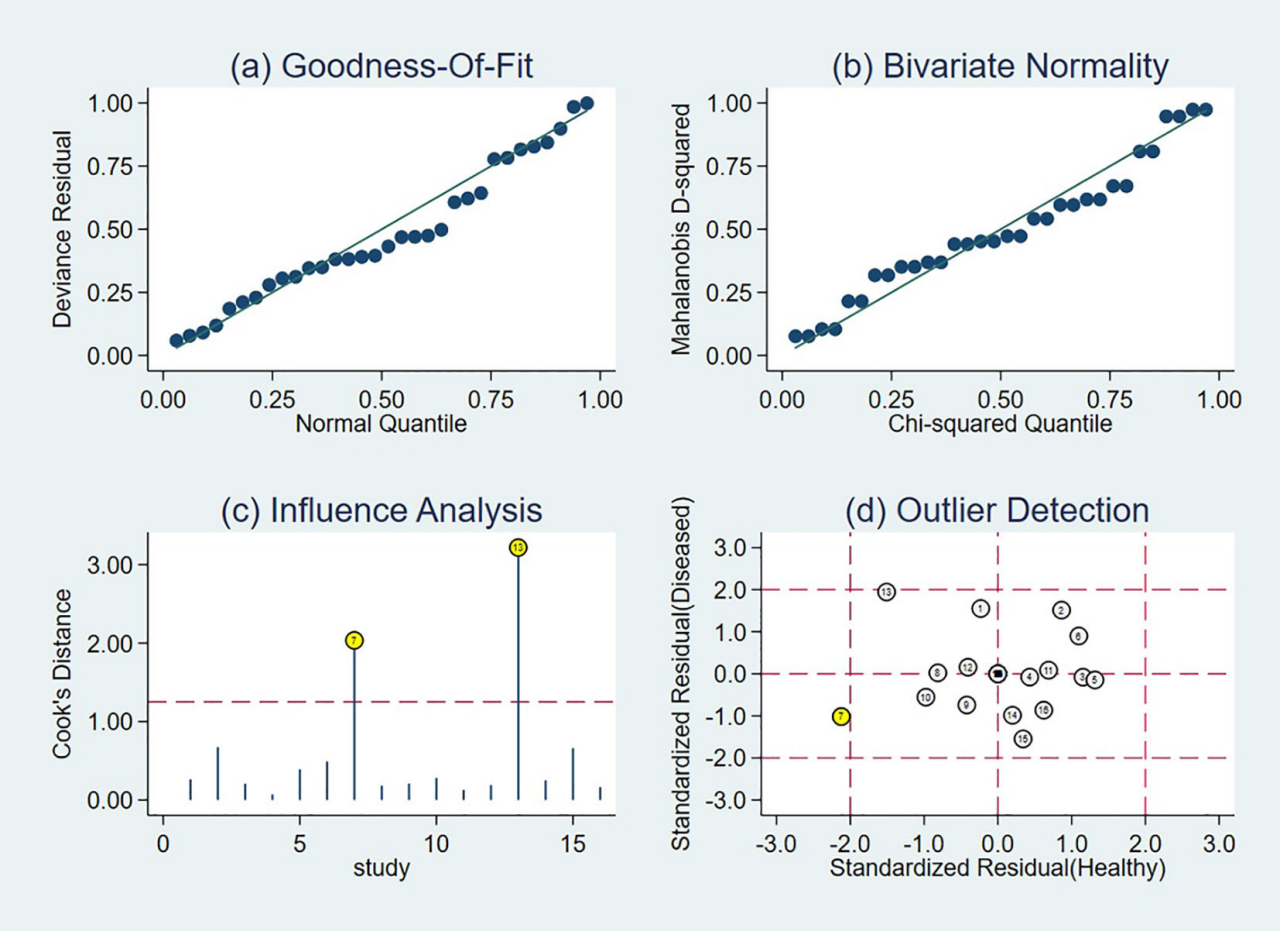
Sensitivity analysis assessing the stability of pooled estimates.

Sensitivity analyses identified two influential studies (18, 19) based on influence diagnostics (**Figure 8**). Upon their concurrent exclusion, the pooled diagnostic estimates were recalculated. The original meta-analytic estimates demonstrated a sensitivity of 0.76 (95% CI: 0.69-0.82) and a specificity of 0.70 (95% CI: 0.59-0.78). Following exclusion, sensitivity remained unchanged at 0.76 (95% CI: 0.70-0.81), while specificity increased to 0.74 (95% CI: 0.65-0.81). A comparative evaluation of CIs revealed substantial overlap between the original and revised estimates, alongside improved precision: the width of the sensitivity CI decreased from 0.13 to 0.11, and that of specificity from 0.19 to 0.16. To further verify the robustness of the results, we additionally conducted a sensitivity analysis by excluding all studies rated as “high risk” in any key domain of QUADAS-2 (n=3, accounting for 9.2% of total patients). After excluding these high-risk studies, the pooled sensitivity was 0.77 (95% CI: 0.67-0.84) and specificity was 0.65 (95% CI: 0.52-0.77). When compared with the original pooled estimates, the 95% CIs of both sensitivity and specificity showed substantial overlap, indicating that excluding high-risk studies did not substantially alter the overall results. These findings collectively reinforce the robustness and stability of the pooled diagnostic estimates across analytic scenarios.

### 3.8 Publication bias

**Figure 9:**
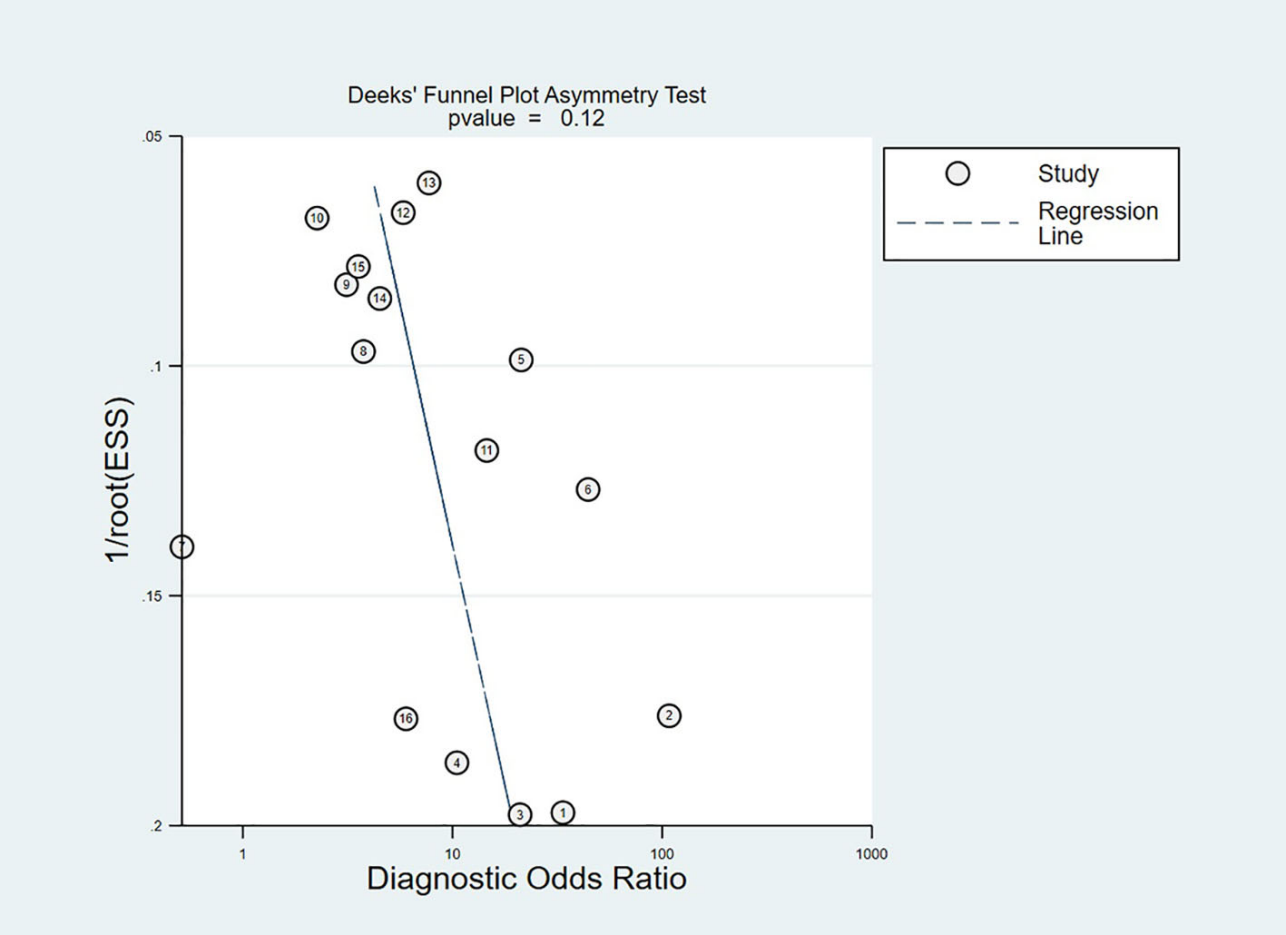
Deeks’ funnel plot.

Publication bias was detected via Deeks’ funnel plot asymmetry test (**Figure 9**), which did not exhibit statistically significant asymmetry (p = 0.12). This non-significant outcome did not indicate no publication bias. The small number of included studies limited the statistical power of the test and may prevent the detection of subtle publication bias. The funnel plot showed no outliers and was relatively symmetrical. These findings indicated the impact of publication bias on pooled estimates was likely small.

## 4 Discussion

This study represents the first meta-analysis systematically elucidating the prognostic performance of presepsin in both sepsis and septic shock, synthesizing data from 16 clinical studies encompassing 2,066 patients. The pooled analysis demonstrates that presepsin possesses a moderate predictive accuracy for mortality, yielding an AUC of 0.80, with a sensitivity of 76% and specificity of 70%. Of particular note, presepsin exhibits markedly higher sensitivity in cohorts with septic shock (90% in comparison to 75% in sepsis), accompanied by a reciprocal decline in specificity (50% versus 77%), suggesting a differential diagnostic utility across varying severities of illness. The considerable heterogeneity observed, primarily attributable to threshold variability and regional disparities, particularly among Korean populations, underscores the pressing need for assay standardization. These findings support the role of presepsin as a pragmatic biomarker for early risk stratification in the critically ill sepsis population.

Our meta-analysis results largely align with those of several individual studies that affirm the prognostic utility of presepsin in sepsis, while also drawing attention to notable discrepancies that merit further discussion. For example, Hassan et al. reported an AUC of 0.824 for in-hospital mortality prediction, which closely approximates our pooled estimate of 0.80 (14). This concordance reinforces the potential of presepsin as a dynamic biomarker, with earlier measurements more accurately capturing the acute phase of the inflammatory response. Similarly, Lee et al. observed that presepsin levels equal to or exceeding 821 pg/mL were linked to a 30-day mortality rate of 33.3%, which aligns with our subgroup analysis indicating higher sensitivity in patients with septic shock relative to those with uncomplicated sepsis (20). This possibly reflects the more profound immune dysregulation observed in septic shock, which drives increased presepsin release (21).

Notably, Koh et al. reported a substantially lower AUC of 0.656, deviating from the pooled estimate derived in our analysis (15). This discrepancy possibly be attributable to differences in study populations. Furthermore, the cutoff value of 1,176 pg/mL employed in their study was derived from a relatively small sample, potentially limiting its generalizability and statistical robustness. In contrast, our meta-analysis, which incorporates data from larger and more diverse cohorts, demonstrates that older patients (aged >65) tend to exhibit marginally lower specificity, possibly due to age-linked alterations in baseline inflammatory markers (22), an observation that is consistent with Koh’s findings.

Geographical differences further contributed to variability in diagnostic performance. For instance, studies conducted in Egypt (e.g., Ali et al., 2016; Hassan et al., 2019) reported superior sensitivity and specificity (14, 23). By contrast, Korean studies (e.g., Kim et al., 2017; Koh et al., 2021) demonstrated comparatively lower specificity (15, 24). Currently, direct evidence fully explaining these regional differences is limited. However, several specific factors should be considered. The studies from Egypt all focused on critically ill patients in the ICU, optimized presepsin cutoffs based on the local population, primarily targeted Gram-negative infections, and excluded confounding from renal failure and cancer, and were mostly prospective in design. In contrast, the studies from Korea primarily included patients in the emergency department or with milder symptoms, used cutoffs not adapted to the local population, had diverse infection sources, and included a high prevalence of comorbid renal failure and cancer, and were mostly retrospective in design. These factors may collectively contribute to the higher sensitivity and specificity in the studies from Egypt.

The study design also exerted a notable influence on observed outcomes. Prospective investigations (e.g., Lee et al., 2022; Juneja et al., 2023) yielded higher sensitivity estimates than retrospective studies, likely due to more rigorous data collection protocols and the timely acquisition of biomarker measurements (18, 20). In contrast, retrospective studies like that by Brodska et al. (2018) faced inherent limitations in accurately capturing baseline presepsin concentrations due to delayed sampling, thereby introducing potential recall and measurement biases (25).

Presepsin, a soluble CD14 subtype (sCD14-ST), derives its prognostic value from its involvement in the biological mechanisms underlying the host immune response to pathogens. CD14 serves as a pivotal receptor within the innate immune system, responsible for recognizing pathogen-associated molecular patterns (PAMPs). Upon stimulation of monocytes and macrophages by microbial components via Toll-like receptor (TLR) signaling pathways during infection, CD14 is cleaved and released into circulation as presepsin (26). This process is intimately linked to the systemic inflammatory response and the onset of organ dysfunction. Accordingly, elevated presepsin levels not only reflect the severity of infection but may also contribute to adverse prognostic outcomes through sustained activation of inflammatory cascades (20, 27). Accumulating evidence indicates that presepsin levels are significantly correlated with the risk of organ failure, like acute kidney injury and acute respiratory distress syndrome, in patients with sepsis. Persistently elevated presepsin concentrations may signify immune dysregulation and failure to control infection (28, 29). Moreover, presepsin demonstrates a relatively stable clearance profile, even in the context of renal impairment, thereby preserving its prognostic reliability in patients with septic shock complicated by multiorgan dysfunction (29, 30). Collectively, these attributes underscore Presepsin’s value as a robust biomarker reflective of the intensity of the host response and the trajectory of disease progression.

Presepsin demonstrates substantial clinical relevance in the management of sepsis and septic shock, and multifaceted utility in critical care settings. It is a biomarker of innate immune activation. Its elevation correlates with disease severity and the extent of organ dysfunction, enabling early stratification of patients at increased risk of mortality (31, 32). Serial measurement of presepsin allows for real-time monitoring of therapeutic efficacy and may serve as a valuable adjunct in antimicrobial stewardship by assessing the adequacy of infection control measures (33, 34). Compared with traditional inflammatory markers, presepsin exhibits enhanced specificity for infectious etiologies, thereby facilitating the differentiation of infectious from non-infectious systemic inflammatory responses and reducing unwarranted antimicrobial administration (35, 36).

While this meta-analysis furnishes comprehensive evidence supporting the prognostic value of presepsin, several limitations merit prudent interpretation of the findings. First, considerable heterogeneity was observed among the included studies, largely attributable to variations in diagnostic thresholds and geographical differences, most notably among Korean cohorts. These discrepancies may reflect differences in assay methodologies, regional pathogen profiles, or clinical practice paradigms, thereby constraining the external validity of the pooled estimates across varied healthcare systems. Second, methodological limitations identified through the QUADAS-2 assessment, including a high or unclear risk of bias in patient selection (six studies), index test implementation (seven studies), and reference standards (eight studies), introduce potential confounders that may compromise the robustness of the results. Third, the unavailability of long-term data hinders the assessment of presepsin level fluctuations and their impact on clinical outcomes. The current body of evidence is predominantly reliant on single-timepoint measurements, whereas serial assessments would offer enhanced prognostic granularity by capturing biomarker kinetics throughout disease progression. Fourth, the paucity of long-term follow-up data limits the assessment of presepsin’s prognostic utility beyond the acute phase, a critical knowledge gap given the substantial burden of chronic morbidity and late mortality among sepsis survivors. Fifth, evidence of publication bias and the predominance of small-scale studies raise concerns regarding potential overestimation of effect sizes, as smaller studies with positive findings are more likely to be published. Lastly, although subgroup analyses identified threshold selection and the geographical region as principal sources of heterogeneity, residual confounding from unmeasured variables, like comorbidities, the timing of antimicrobial initiation, or strategies for organ support, along with methodological deficiencies in the primary studies, may further influence the prognostic performance of presepsin.

Future research should prioritize large-scale, multicenter investigations employing standardized assay protocols to validate presepsin’s prognostic thresholds and address population- and assay-related heterogeneity. Integration of serial presepsin measurements with time-series analyses may enhance dynamic risk stratification by delineating biomarker trajectories in response to therapeutic interventions. Combining presepsin with complementary biomarkers, like PCT or lactate, and established clinical scoring systems (e.g., qSOFA, APACHE II), potentially through machine learning-based predictive models, may augment prognostic accuracy beyond the limitations of single biomarkers. The advent of microfluidic biosensor technology offers promising avenues for real-time monitoring, particularly in resource-constrained environments (37). Additionally, extended follow-up studies are warranted to elucidate presepsin’s prognostic relevance in the post-acute phase, including its utility in predicting post-sepsis syndrome and long-term outcomes.

Future research should prioritize large-scale multicenter studies with standardized protocols to validate presepsin’s prognostic thresholds and address heterogeneity across populations and assay platforms. Serial presepsin measurements integrated with time-series analyses could enhance dynamic risk stratification by capturing biomarker trajectories relative to therapeutic responses. Combining presepsin with complementary biomarkers (e.g., PCT, lactate) and clinical scores (qSOFA, APACHE II) through machine learning-based models possibly improves predictive accuracy beyond single-marker limitations. Emerging technologies like microfluidic biosensors enable real-time monitoring, particularly valuable in resource-limited settings. Additionally, extended follow-up studies are needed to evaluate presepsin’s role in predicting post-sepsis syndrome and long-term outcomes.

## 5 Conclusion

This meta-analysis substantiates presepsin as a promising prognostic biomarker in sepsis and septic shock, demonstrating the consistent potential for mortality risk stratification across diverse clinical settings. Grounded in its pathophysiological association with innate immune dysregulation and characterized by its rapid detectability, presepsin holds significant promise for clinical integration as a decision-support tool, facilitating early identification of high-risk patients and enabling the timely allocation of critical care resources.

## Data Availability

The original contributions presented in the study are included in the article/**Supplementary Material**. Further inquiries can be directed to the corresponding author.
