# Supplementary material for: Prognostic value of presepsin in sepsis and septic shock: a meta-analysis
Source: Front Immunol. 2025 Oct 15;16:1680877. doi: 10.3389/fimmu.2025.1680877 (PMC12568590; doi:10.3389/fimmu.2025.1680877)
Supplement: Supplementary file 1 [file Table1.docx]

**Supplementary Materials**

**Supplementary Table S1** Search Strategies

| Pubmed | | |
| --- | --- | --- |
| # | Query | Results |
| 1 | Sepsis[MeSH Terms] | 146,315 |
| 2 | Shock, Septic[MeSH Terms] | 25,567 |
| 3 | "Blood Poisoning*"[Title/Abstract] OR "Bloodstream Infection*"[Title/Abstract] OR "Endotoxin Shock*"[Title/Abstract] OR "Pyaemia*"[Title/Abstract] OR "Pyemia*"[Title/Abstract] OR "Pyohemia*"[Title/Abstract] OR "sepsis"[Title/Abstract] OR "septic disease"[Title/Abstract] OR "septic* shock"[Title/Abstract] OR "Septicemia*"[Title/Abstract] OR "Toxic Shock"[Title/Abstract] | 177,963 |
| 4 | #1 OR #2 OR #3 | 245,167 |
| 5 | "presepsin protein, human" [Supplementary Concept] | 300 |
| 6 | "CD14 Antigen*"[Title/Abstract] OR "Lipoglycan Receptor"[Title/Abstract] OR "Lipopolysaccharide Receptors"[Title/Abstract] OR "LPS Receptor"[Title/Abstract] OR "presepsin"[Title/Abstract] OR "sCD14"[Title/Abstract] OR "Soluble CD14"[Title/Abstract] | 3,174 |
| 7 | #5 OR #6 | 3,181 |
| 8 | #4 AND #7 | 656 |

| Embase | | |
| --- | --- | --- |
| # | Query | Results |
| 1 | 'sepsis'/exp | 363751 |
| 2 | 'septic shock'/exp | 76503 |
| 3 | 'blood poisoning*':ab,kw,ti OR 'bloodstream infection*':ab,kw,ti OR 'endotoxin shock*':ab,kw,ti OR 'pyaemia*':ab,kw,ti OR 'pyemia*':ab,kw,ti OR 'pyohemia*':ab,kw,ti OR 'sepsis':ab,kw,ti OR 'septic disease':ab,kw,ti OR 'septic* shock':ab,kw,ti OR 'septicemia*':ab,kw,ti OR 'toxic shock':ab,kw,ti | 272056 |
| 4 | #1 OR #2 OR #3 | 434051 |
| 5 | 'presepsin'/exp | 404 |
| 6 | 'cd14 antigen*':ab,kw,ti OR 'lipoglycan receptor':ab,kw,ti OR 'lipopolysaccharide receptors':ab,kw,ti OR 'lps receptor':ab,kw,ti OR 'presepsin':ab,kw,ti OR 'scd14':ab,kw,ti OR 'soluble cd14':ab,kw,ti | 4759 |
| 7 | #5 OR #6 | 4800 |
| 8 | #4 AND #7 | 989 |

| Cochrane Library | | |
| --- | --- | --- |
| # |  |  |
| 1 | MeSH descriptor: [Sepsis] explode all trees | 6497 |
| 2 | MeSH descriptor: [Shock, Septic] explode all trees | 1415 |
| 3 | ('Blood Poisoning*' OR 'Bloodstream Infection*' OR 'Endotoxin Shock*' OR 'Pyaemia*' OR 'Pyemia*' OR 'Pyohemia*' OR 'sepsis' OR 'septic disease' OR 'septic* shock' OR 'Septicemia*' OR 'Toxic Shock'):ab,kw,ti | 18945 |
| 4 | #1 OR #2 OR #3 | 20779 |
| 5 | MeSH descriptor: [Lipopolysaccharide Receptors] explode all trees | 174 |
| 6 | ('CD14 Antigen*' OR 'Lipoglycan Receptor' OR 'Lipopolysaccharide Receptors' OR 'LPS Receptor' OR 'presepsin' OR 'sCD14' OR 'Soluble CD14'):ab,kw,ti | 1016 |
| 7 | #5 OR #6 | 1016 |
| 8 | #4 AND #7 | 136 |

| Web of Science | | |
| --- | --- | --- |
| # | Query | Results |
| 1 | TS=((Blood Poisoning*) OR (Bloodstream Infection*) OR (Endotoxin Shock*) OR (Pyaemia*) OR (Pyemia*) OR (Pyohemia*) OR (sepsis) OR (septic disease) OR (septic* shock) OR (Septicemia*) OR (Toxic Shock)) | 228653 |
| 2 | TS=((CD14 Antigen*) OR (Lipoglycan Receptor) OR (Lipopolysaccharide Receptors) OR (LPS Receptor) OR (presepsin) OR (sCD14) OR (Soluble CD14)) | 52812 |
| 3 | #1 AND #2 | 6418 |

**Supplementary Table S2** QUADAS-2 Quality Assessment

|  |  | Abdelshafey 2021 | Ali 2016 | Baik 2022 | Brodska 2018 | Drăgoescu 2021 | Hassan 2019 | Juneja 2023 | Kim 2017 | Koh 2021 | Lee 2022 | Narendra 2022 | Park 2021 | Ren 2024 | Wen 2019 | Wu 2023 | Yang 2024 |
| --- | --- | --- | --- | --- | --- | --- | --- | --- | --- | --- | --- | --- | --- | --- | --- | --- | --- |
| Patient selection | Question 1 | Yes | No | Unclear | Yes | Yes | Yes | No | Yes | Yes | Yes | No | Yes | Yes | No | Yes | Yes |
|  | Question 2 | Yes | No | No | Yes | Yes | Yes | No | Yes | No | Yes | No | Yes | Yes | No | Yes | Yes |
|  | Question 3 | Yes | Unclear | No | Yes | Yes | Yes | Yes | Yes | Yes | Yes | Yes | Yes | Yes | Yes | Yes | Yes |
|  | Risk of bias | Low | Unclear | Unclear | Low | Low | Low | Unclear | Low | Unclear | Low | Unclear | Low | Low | Unclear | Low | Low |
|  | Concerns regarding applicability | Low | Unclear | High | Low | Unclear | Low | Low | Low | Low | Unclear | Low | Low | Low | Low | Low | Low |
| Index test(s) | Question 1 | Yes | No | Unclear | Unclear | Yes | Yes | No | Yes | No | Yes | No | Yes | Yes | Yes | Unclear | Yes |
|  | Question 2 | Yes | No | No | Yes | Yes | Yes | Yes | Yes | Yes | Yes | Yes | Yes | Yes | Yes | Yes | Yes |
|  | Risk of bias | Low | High | Unclear | Unclear | Low | Low | Unclear | Low | Unclear | Low | Unclear | Low | Low | Unclear | Unclear | Low |
|  | Concerns regarding applicability | Low | High | Unclear | Low | Low | Low | Low | Low | Low | Low | Low | Unclear | Low | Low | Low | Low |
| Reference standard | Question 1 | Yes | Yes | No | No | Yes | Yes | Yes | No | Yes | No | Yes | Yes | Yes | No | No | Yes |
|  | Question 2 | Yes | Yes | Unclear | No | Yes | Yes | Yes | Yes | Yes | No | No | Yes | No | No | Yes | Yes |
|  | Risk of bias | Low | Low | Unclear | High | Low | Low | Low | Unclear | Low | High | Unclear | Low | Unclear | High | Unclear | Low |
|  | Concerns regarding applicability | Low | Low | Low | High | Unclear | Low | Low | Low | Unclear | Unclear | High | Low | Unclear | Low | Low | Unclear |
| Flow and timing | Question 1 | Yes | No | Yes | Yes | Yes | Yes | Yes | Yes | Yes | Yes | Yes | Yes | Yes | Yes | Yes | Yes |
|  | Question 2 | Yes | No | Yes | Yes | Yes | No | No | No | Yes | No | Yes | Yes | No | No | Yes | No |
|  | Question 3 | Yes | No | Yes | Yes | Yes | Yes | Yes | Yes | Yes | Yes | Yes | Yes | Yes | Yes | Yes | Yes |
|  | Risk of bias | Low | High | Low | Low | Low | Unclear | Unclear | Unclear | Low | Unclear | Low | Low | Unclear | Unclear | Low | Unclear |
